# Estimation of Daily Ground-Received Global Solar Radiation Using Air Pollutant Data

**DOI:** 10.3389/fpubh.2022.860107

**Published:** 2022-04-04

**Authors:** Xinshuo Zhang, Mengli Zhang, Yong Cui, Ying He

**Affiliations:** ^1^Faculty of Architecture and Urban Planning, Chongqing University, Chongqing, China; ^2^Chongqing Real Estate Trading Centre, Chongqing, China; ^3^Key Laboratory of New Technology for Construction of Cites in Mountain Area, Chongqing University, Chongqing, China

**Keywords:** global solar radiation, prediction models, air quality index, air pollutants, meteorological factors

## Abstract

Ground-received solar radiation is affected by several meteorological and air pollution factors. Previous studies have mainly focused on the effects of meteorological factors on solar radiation, but research on the influence of air pollutants is limited. Therefore, this study aimed to analyse the effects of air pollution characteristics on solar radiation. Meteorological data, air quality index (AQI) data, and data on the concentrations of six air pollutants (O_3_, CO, SO_2_, PM_10_, PM_2.5_, and NO_2_) in nine cities in China were considered for analysis. A city model (model-C) based on the data of each city and a unified model (model-U) based on national data were established, and the key pollutants under these conditions were identified. Correlation analysis was performed between each pollutant and the daily global solar radiation. The correlation between O_3_ and daily global solar radiation was the highest (*r* = 0.575), while that between SO_2_ and daily global solar radiation was the lowest. Further, AQI and solar radiation were negatively correlated, while some pollution components (e.g., O_3_) were positively correlated with the daily global solar radiation. Different key pollutants affected the solar radiation in each city. In Shenyang and Guangzhou, the driving effect of particles on the daily global solar radiation was stronger than that of pollutants. However, there were no key pollutants that affect solar radiation in Shanghai. Furthermore, the prediction performance of model-U was not as good as that of model-C. The model-U showed a good performance for Urumqi (*R*^2^ = 0.803), while the difference between the two models was not particularly significant in other areas. This study provides significant insights to improve the accuracy of regional solar radiation prediction and fill the gap regarding the absence of long-term solar radiation monitoring data in some areas.

## Introduction

Solar radiation is one of the main energy sources of the Earth–atmosphere system and drives atmospheric motion (1). In recent decades, the solar radiation trends in most regions worldwide have been decreasing. For instance, in the 1990's, a global dimming phenomenon, which was more evident in large cities, was observed (2–4). The reduction in solar radiation caused by global dimming can change the climate and reduce the surface temperature of Earth (5, 6). Long-term and accurate assessments of the amount of solar radiation reaching the Earth's surface is important for determining the energy budget of the Earth–atmosphere system, and to study climate change, evaluate solar radiation patterns, and develop and utilize solar energy resources (7–9). And high concentration of particulate matters in air reduced the amount of solar radiation that can reach the earth (10, 11). Appropriate lighting is also conducive to creating a more liveable urban environment and encouraging more citizens to participate in outdoor activities, such as outdoor walking (12).

The regional estimation of solar radiation has shifted from measurements using expensive and highly maintained instruments to model calculations (13, 14). Various empirical models, for example, sunshine-based models (15–18) and temperature-based models (19–21) are commonly used due to their simple operation and low computational costs (22–26). Angstrom (27) and Prescott (28) first proposed a simple solar radiation calculation model using average clear-sky daily global radiation data and the sunshine duration of the selected study area. Many subsequent studies further added expressions (e.g., quadratic, cubic, square root, logarithm, exponent, and idempotent) to the Angström–Prescott model (16, 22).

Further, various meteorological factors, such as precipitation and relative humidity, have been applied to improve the model accuracy (29–33). In addition to empirical models, machine learning models have been used for global solar radiation prediction to address non-linear and multidimensional relationships between solar radiation and meteorological factors in noisy environments (34–38). Fan et al. (39) indicated that by using vapor pressure deficit and relatively humidity, daily global solar radiation could be estimated more accurately in South China, which experiences a humid subtropical or tropical climate. Zhang et al. (31) reviewed and compared multiple models in terms of time scale and estimation type, and proved that sunshine-based and artificial neural network models exhibited similar performances in estimating monthly average and daily global radiation. The advantage of regression model is that the understanding and interpretation of the model are very intuitive. Artificial neural network model belongs to black box, which is difficult to understand the internal mechanism.

With expanding industrialization and the increasing proliferation of cars, especially in developing countries, the existing issue of air pollution is bound to further aggravate. Some researchers began to consider the impact of air pollution on solar radiation. Air pollution can change the amount of total solar radiation reaching the ground surface (40). Further, suspended particles capable of scattering and absorbing radiation can weaken the ground solar radiation. Elminir (41) concluded that air pollution reduced the total ground radiation in Egypt by 9.3–22% under clear sky conditions. Moreover, Fu and Dan (42) reported that an increased atmospheric aerosol concentration significantly affected the number of sunshine hours and the proportion of scattered radiation. Therefore, considering the impact of air pollution when estimating solar radiation estimation is important. Considering the impact of air pollution, the previous empirical solar radiation model may be difficult to meet the needs of solar energy utilization. Some researchers have studied the impacts of air pollution index (API) or air quality index (AQI) on solar radiation prediction (43–45). Furlan et al. (46) introduced a new regression model that considered the effects of air pollution and cloud cover on hourly diffuse solar radiation data. Their results showed that their model performed better than previously developed models. Further, Janjai et al. (47) proposed a semi-empirical model to estimate clear sky global and direct normal solar irradiances in Thailand. The model included physical parameters, such as aerosol optical properties and perceptible water, and performed better than previous empirical models. The validation of solar radiation data acquired from several cities showed that the new daily diffuse solar radiation (NDDSR) model and its modified version were applicable to various regions. Zhao et al. (48) and Suthar et al. (49) established linear, exponential, and logarithmic empirical models using data from China and India, and their results showed that inclusion of air pollution can improve the prediction accuracy of models. Moreover, Yao et al. (50) developed a new method using AQI and solar radiation data of 55 years measured in Beijing to modify the existing NDDSR model.

Although many studies have explored the impacts of air pollution on solar radiation, most focused only on the impact of AQI on solar radiation, and neglected the regional differences in the impacts of different pollutants on solar radiation. Presently, air quality monitoring mainly assesses the mass concentrations of PM_2.5_, PM_10_, NO_2_, SO_2_, CO, and O_3_ (9, 51–53). The standardization of urban air quality monitoring procedures can facilitate the use of measured data on different pollutants and the corresponding solar radiation for further analysis.

As few studies have analyzed the impact of air pollutants on solar radiation estimation, the present study filled this research gap. This study aimed to analyse the correlation characteristics between air pollution and daily global solar radiation. Data on various meteorological factors, AQI, and six air pollutants (O_3_, CO, SO_2_, PM_10_, PM_2.5_, and NO_2_) were considered from nine Chinese cities from 2015 to 2020. Further, the influence mechanism of air pollutants on daily global solar radiation was analyzed using stepwise regression. Moreover, a unified model (model-U) based on national data and city model (model-C) based on the data of each city were obtained through regression analysis, and the key pollutants that significantly affected solar radiation were identified. In particular, this study aimed: (1) to explore the correlation between different meteorological parameters, air pollution parameters, and solar radiation; (2) to establish a solar radiation model to analyse the influencing characteristics of pollutant parameters on solar radiation in each city; (3) to propose a sunshine pollution model-U with universal applicability in China and compare its performance with model-C; and (4) to extract the key pollutants for model-U and each model-C. The characteristics of the impact of pollutants on solar radiation in different cities were revealed and regional solar radiation prediction strategies were formulated.

## Materials and Methods

### Study Area

**Figure 1:**
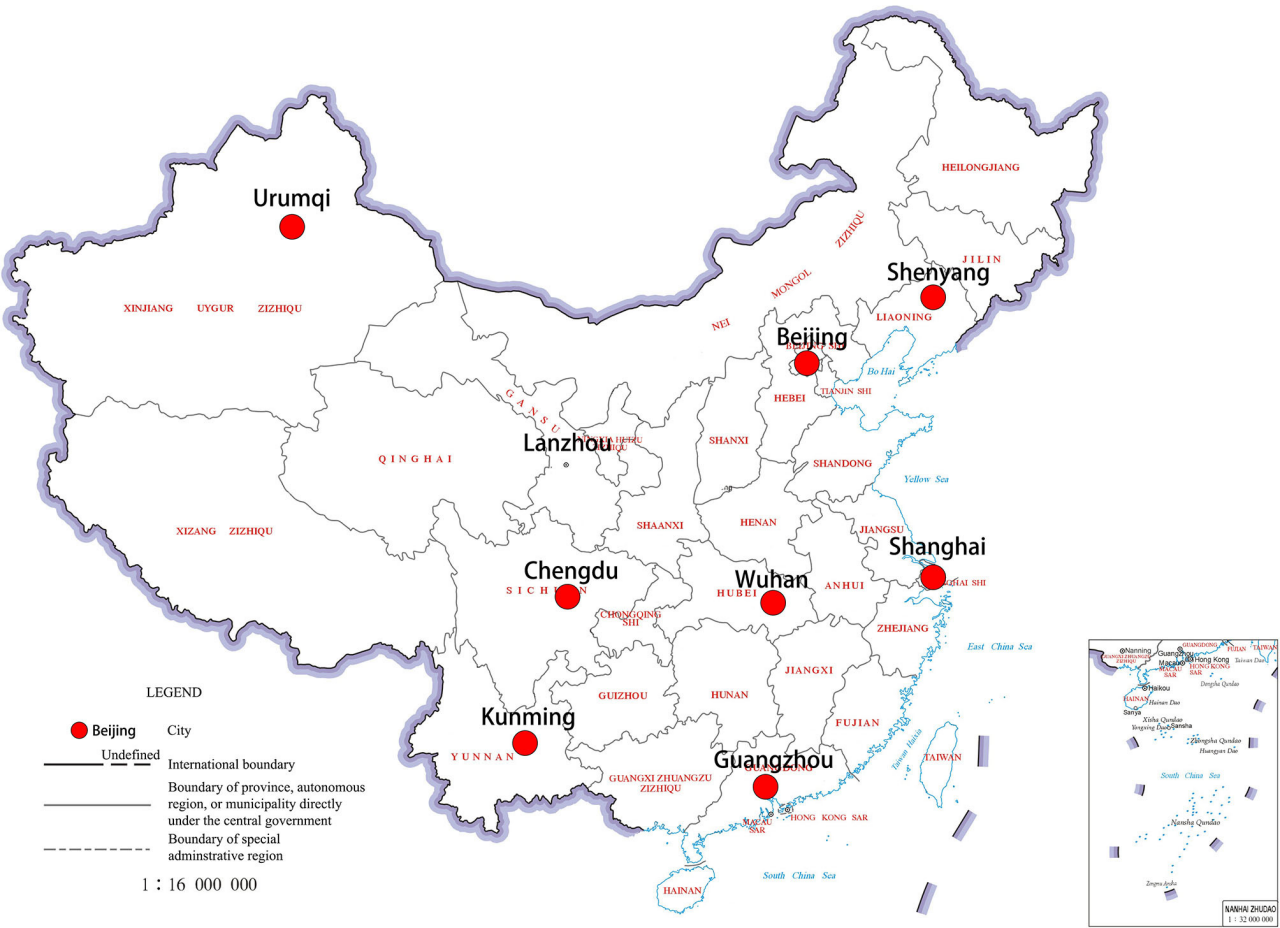
The geographic location of the study area.

The study area included the following nine Chinese cities: Urumqi, Lanzhou, Shenyang, Beijing, Chengdu, Kunming, Wuhan, Shanghai, and Guangzhou, with varying geographical locations (Urumqi and Lanzhou–northwest, Shenyang and Beijing–northeast, Guangzhou–southeast coast, Chengdu and Kunming–southwest, Wuhan–central China, and Shanghai–eastern coast) (Figure 1). Further, Shanghai, Guangzhou, Wuhan, Kunming, and Chengdu experience a subtropical monsoon climate, Beijing experiences a temperate monsoon climate, Shenyang experiences a temperate semi humid continental climate, and Urumqi and Lanzhou experience a temperate continental climate. Lanzhou and Shanghai have the highest (1,874.4 m) and lowest altitude (5.5 m), respectively. Further, the annual average temperature of Harbin, Urumqi, Lanzhou, and Shenyang is relatively low. The lowest annual average relative humidity is observed in Beijing (51.14%). Moreover, the annual average sunshine hours are the lowest in Chengdu (2.86 h), while Guangzhou has the highest annual average precipitation.

The selected nine cities suffer from air pollution, particularly with high concentrations of inhalable particulate matter and haze. In Urumqi and Beijing, the annual average AQI is 96.59 and 87.33, respectively. The API in Kunming and Guangzhou is generally low, while the annual average AQI is 54.83 and 54.09, respectively. Urumqi has the highest levels of inhalable particulate matter and NO_2_ among the nine cities, while Shenyang has the highest SO_2_ concentration. Further, the O_3_ concentration is the highest in Lanzhou, while the CO concentration in all cities is similar. Different pollution sources cause differences in the pollutant characteristics in different cities.

In terms of solar radiation, the average annual total solar radiation in Urumqi is the highest (6,123 MJ/m^2^). However, due to a higher proportion of rain and fog and less sunny days, the average annual total solar radiation in Chengdu is only 3,854 MJ/m^2^.

### Data Sources

Meteorological data, including daily observation values of average relative humidity (%), daily maximum temperature (°C), minimum temperature (°C), sunshine duration (h), and precipitation (mm), were acquired from the National Meteorological Information Center (http://data.cma.cn/). The acquired real time data were quality controlled and the availability of all elements exceeded 99.9%, with the percentage of correct data being 100%.

Solar radiation data were acquired from the National Meteorological Information Center. The dataset was mainly developed based on the HYBRID/MLWT2 model. Compared with the measured value, the average error of the product was −0.1 W/m^2^, relative error was −0.04%, and correlation coefficient was 0.98 (http://data.cma.cn/Market/Detail/code/RADI_CHN_MUL_HOR/type/0.html).

The air quality data (comprising all compositional information) were obtained from the national urban air quality real-time release platform of China Environmental Monitoring Station. The dataset provides data on AQI and six air pollutants (PM_2.5_, PM_10_, SO_2_, NO_2_, CO, and O_3_; http://www.cnemc.cn/).

According to previous studies (27, 28), actual daily sunshine hours *n*, daily potential sunshine hours *N*, daily extra-terrestrial solar radiation *Q*_0_, daily minimum temperature *T*_min_, relative humidity *R*_*h*_, and maximum temperature *T*_max_ are typically the most influential input parameters to predict global solar radiation. Sunshine rate is the ratio of the actual daily sunshine hours and the daily potential sunshine hours. In addition to the daily maximum and minimum temperatures, some researchers used daily temperature difference Δ*T* or the logarithm of temperature difference ln(Δ*T*) in their empirical equations (29, 54, 55). Wind and urban heat island effect are also one of the influencing factors of air pollution accumulation and photochemical reaction. Wind can affect the density and spatial distribution of air pollutants, and also affects the speed of photochemical reaction (12, 56, 57). Due to the existence of ground buildings, the wind also changes greatly in a small size range. It is difficult for traditional weather stations to achieve the required measurement density (58).

In this study, 14 initial independent variables were selected, including one radiation variable (daily extra-terrestrial solar radiation *Q*_0_), seven pollution factors (AQI, O_3_, CO, SO_2_, PM_10_, PM_2.5_, and NO_2_), and six meteorological variables [ratio of the daily actual sunshine hours and the daily potential sunshine hours, relative humidity *R*_*h*_, daily minimum temperature *T*_min_, daily maximum temperature *T*_max_, daily temperature difference Δ*T*, and logarithm of temperature difference ln(Δ*T*)]. Four different temperature representation methods were selected to determine the most suitable representation method for the model.

Further, the daily average values of all air pollution data from 2015 to 2020 were calculated and associated with the acquired meteorological data and daily global solar radiation data. Incomplete datasets were deleted; additionally, to exclude the influence of thick cloud cover, only zero precipitation data were selected. The final data volume of each city was as follows: Urumqi−1,340 groups, Lanzhou−1,577 groups, Shenyang−1,603 groups, Beijing−1,599 groups, Chengdu−1,173 groups, Kunming−1,356 groups, Wuhan−1,077 groups (without 2018 data), Shanghai−1,226 groups, and Guangzhou−1,169 groups. Differences in the amount of data could affect the model accuracy.

### Methods

**Figure 2:**
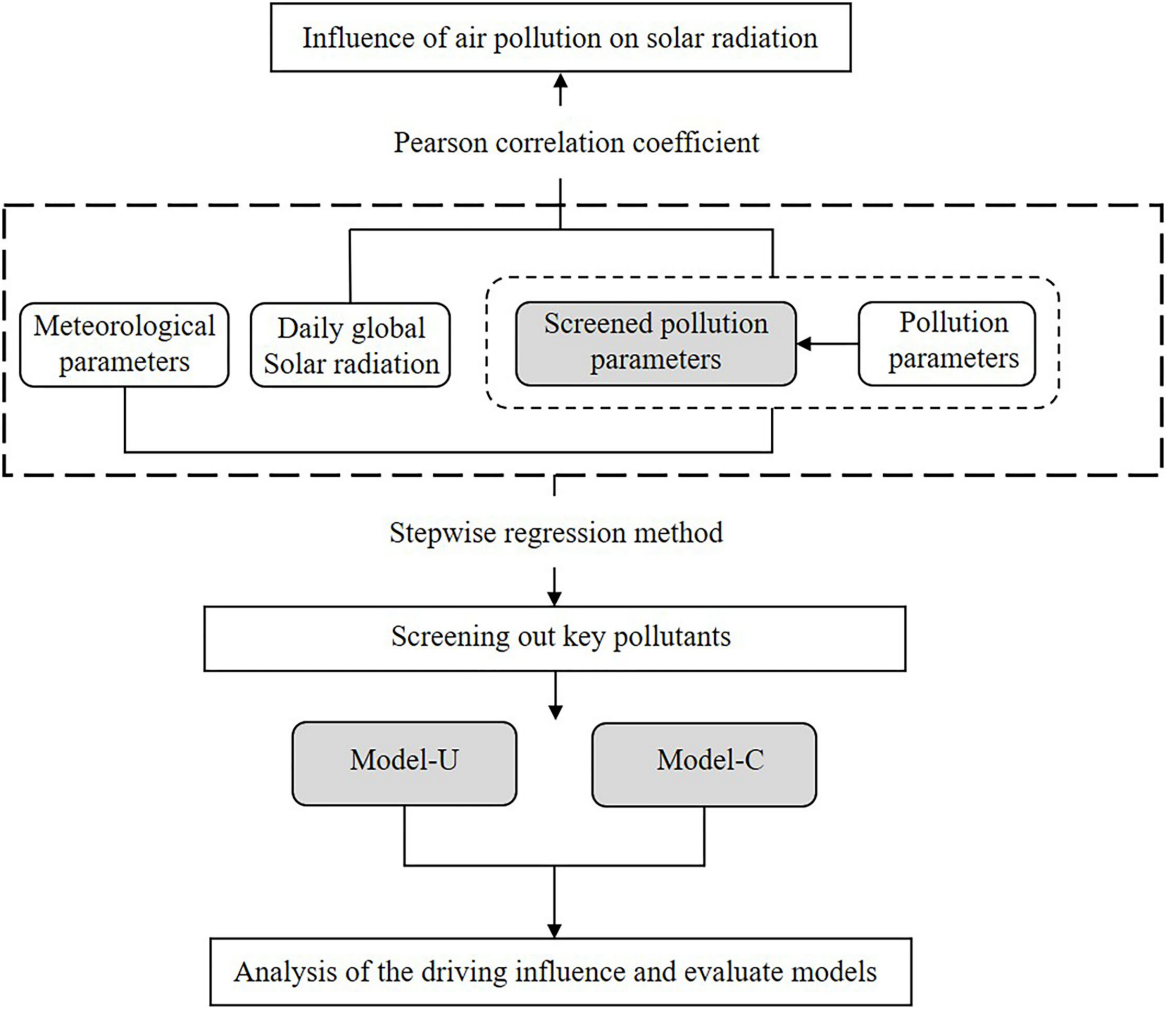
Structure and technical framework of the study.

Figure 2 presents the method for analyzing the driving effect of air pollution on solar radiation. Pearson's correlation was applied to determine the relation between daily global solar radiation and the air pollution parameters. Further, linear regression analysis was used to investigate the driving influence of air pollution factors on daily global solar radiation and determine the key pollutants that have strong effects on daily global solar radiation. Nine city models (model-C) based on the data of each city and a unified model (model-U) based on national data using comprehensive city data were established using stepwise regression analysis. The comparative analysis of model-C and model-U was conducted to discuss the regional differences in the impact of air pollution on solar radiation.

#### Stepwise Regression Analysis Model

The prediction model was established using stepwise regression, which introduced the variables that affect daily global solar radiation step-by-step, determined the variables most significantly affecting daily global solar radiation each time, tested the existing variables in the equation, and eliminated the variables determined to be insignificant. Finally, neither new variables were introduced nor were old variables deleted. The calculation formula is:


$$\begin{array}{l} Y\;=\;\beta_{0}\;+\;\beta_{i}X_{i}\;+\;\varepsilon ,i\;=\;1,\cdots \;,p \end{array}$$

where *X*_*i*_ is the independent variable affecting solar radiation and *Y* is the dependent variable. The F-test statistic value of the corresponding regression coefficient of *X*_*i*_ is calculated as given below, and recorded as $F_{1}^{\left(1\right)},\cdots \;,F_{p}^{\left(1\right)}$, with the maximum value given as $F_{i_{1}}^{\left(1\right)}$.


$$\begin{array}{l} F_{i_{1}}^{\left(1\right)}\;=\;\max\left\{\overset{\left(1\right)}{\underset{1}{F}},\cdots \;,F_{p}^{\left(1\right)}\right\} \end{array}$$

At a significance level α, the corresponding critical value was recorded as $F_{1}^{\left(1\right)}$, $F_{i_{2}}^{\left(1\right)}\geq F^{\left(2\right)}$; subsequently, *X*_*i*_2__ was introduced into the regression model and *I*_1_ was added to index set.

This method was repeated, and an independent variable that had not been introduced into the regression model was selected each time until no variable was left to be introduced.

#### Model Assessment and Statistical Error Analysis

Coefficient of determination (*R*^2^), root mean square error (RMSE), mean absolute error (MAE) and mean deviation error (MBE) were used to evaluate the accuracy and performance of the models. To compare model performance, Lin's Concordance Correlation Coefficient (LCCC) (59) were used, which were calculated as follows:


$$\begin{array}{l} LCCC\;=\;\frac{2s_{xy}}{s_{x}^{2}\;+\;s_{y}^{2}+{\left(\bar{x}\;-\;\bar{y}\right)}^{2}} \end{array}$$

where *x*_*i*_ and *y*_*i*_ are the measured and predicted daily global solar radiation; $\bar{x}$ and $\bar{y}$ are the means for *x*_*i*_ and *y*_*i*_;and $s_{x}^{2}$ and $s_{y}^{2}$ are the corresponding variances and,

$$\begin{array}{l} s_{xy}\;=\;\frac{1}{n}\overset{n}{\underset{i\;=\;1}{\sum }}\left(x_{i}\;-\;\bar{x}\right)\left(y_{i}\;-\;\bar{y}\right) \end{array}$$

According to Zhao et al. (60), LCCC = 1 indicates perfect agreement. LCCC larger than 0.9 means excellent agreement, and the value ranging from 0.80 to 0.90 shows good agreement. The moderate agreement is achieved when LCCC values are between 0.65 and 0.80, while the values <0.65 mean poor agreement.

## Results

### Comparative Analysis of Air Pollution and Solar Radiation

**Figure 3:**
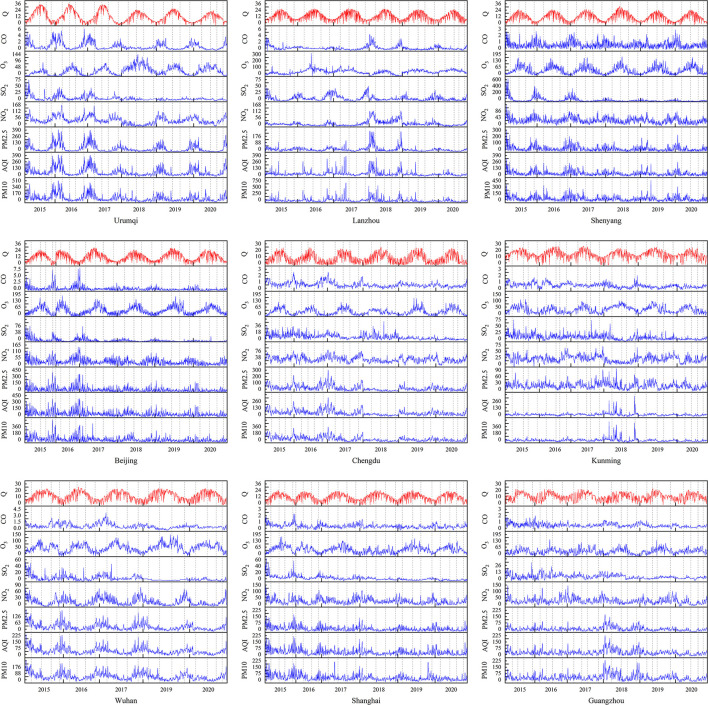
Daily global solar radiation and pollution parameters from 2015 to 2020 in nine cities (Wuhan lacks 2018 data).

Figure 3 shows the daily global solar radiation and pollutant parameters of the selected nine cities from 2015 to 2020. The daily global solar radiation showed evident periodicity, with low and high values observed in winter and summer, respectively. The pollutant concentrations in Urumqi, Shenyang, Beijing, and Wuhan changed periodically, which was not evident in other cities. The AQI levels of Urumqi and Beijing were initially high in 2015 and 2016, and then decreased. In Lanzhou, Kunming, and Guangzhou, the AQI levels in 2018 were higher than those in other years. Moreover, in Shenyang and Shanghai, AQI levels did not have considerable yearly variations, and no evident fluctuations were observed. The variations in the AQI level in each city may be related to the industrial development and population density of each city. Furthermore, AQI level as well as CO, NO_2_, SO_2_, PM_10_, and PM_2.5_ were negatively correlated with daily total solar radiation. Contrastingly, O_3_ showed a significant periodic change, similar to the daily total solar radiation. This indicated that the influence of various pollutants and comprehensive AQI on global daily solar radiation was not consistent.

### Analysis of the Influence of Air Pollution on Solar Radiation

#### Correlation Analysis Between Solar Radiation and Air Pollution

**Figure 4:**
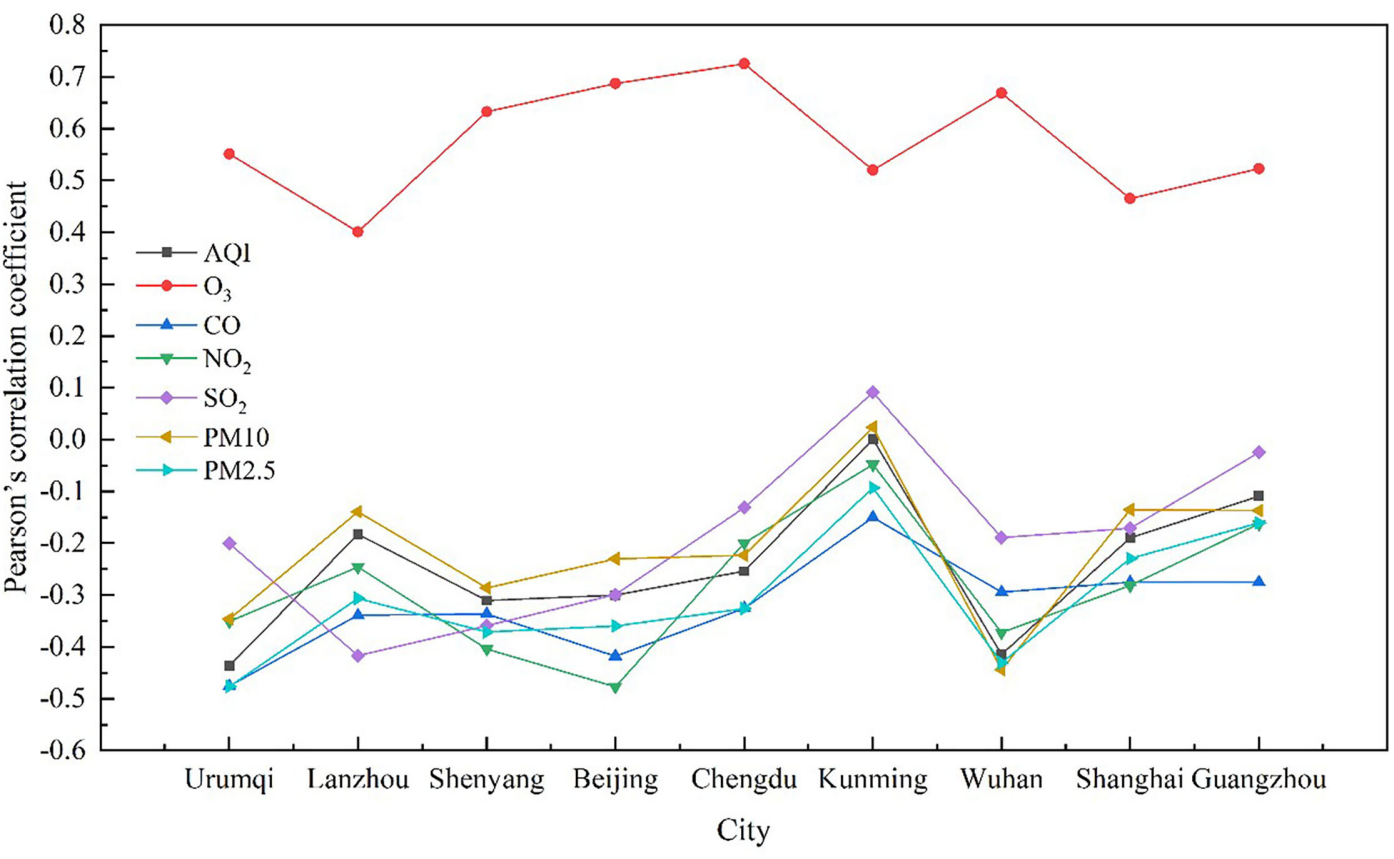
Correlation coefficients between air pollution parameters and daily global solar radiation in the nine cities from 2015 to 2020.

Figure 4 shows the correlation coefficients between daily global solar radiation and air pollution in the nine cities during 2015–2020. The correlation between any single pollutant and daily global solar radiation differed. O_3_ and daily global solar radiation showed the highest correlation (*r* = 0.575), while the correlation between SO_2_ and daily global solar radiation was the lowest. AQI, CO, NO_2_, PM_10_, and PM_2.5_ were negatively correlated with daily global solar radiation. Further, the correlation coefficients between various pollution parameters and daily global solar radiation showed spatial heterogeneity. SO_2_ showed a negative correlation in all areas, except Kunming, and O_3_ showed a positive correlation with daily global solar radiation in all nine cities. Except for O_3_, the correlation of all other pollutants with daily global solar radiation was significantly weaker in Kunming than that in other areas. This could be attributed to the climatic conditions of Kunming, which facilitates the diffusion of pollutants. In addition, the air quality in this city was excellent due to long-term uniform precipitation. In 2020, the annual rate of air quality in Kunming was 100%. Moreover, the number of excellent days was 203 and the number of good days was 163.

#### Influence of Air Pollution on Daily Global Solar Radiation and the Selection of Key Pollutants

Table 1 shows the regression analysis results of the selected factors for the model to explain daily global solar radiation. Based on the regression analysis results, the pollution factors with a strong effect on daily global solar radiation were labeled as key pollutants for further investigation. Subsequently, daily extra-terrestrial solar radiation *Q*_0_ and sunshine rate were the most important factors affecting solar radiation, and were always selected as the top two elements of model-U and model-C. Among these, *Q*_0_ was related to the local latitude, while the sunshine rate represented local sunshine conditions. In addition to the above two factors, temperature contributed the most to the model in Beijing, Wuhan, Shanghai, and Guangzhou. During the selection of modeling elements, four temperature types were provided. According to the regression analysis results, model-U contained nine variables, and their order of contribution was *Q*_0_, the ratio of the daily actual sunshine hours and the daily potential sunshine hours, *R*_*h*_, *T*_min_, *T*_max_, O_3_, NO_2_, and SO_2_. Further, the results showed that *T*_min_ had the greatest contribution to model-U in the four temperature representations, while ln(Δ*T*) and Δ*T* had lower contributions. *T*_max_ and *T*_min_ combined contributed more to the model than ln(Δ*T*) alone; additionally, *T*_min_ had the highest impact on the solar radiation, and approximately half of the total sites in model-C showed the same results.

**Table 1:** Elements of the city models and ranking of their contributions.

**Rank**	**Model-C**									**Model-U**
	**Urumqi**	**Lanzhou**	**Shenyang**	**Beijing**	**Chengdu**	**Kunming**	**Wuhan**	**Shanghai**	**Guangzhou**	
1	*Q*_0_	Sunshine rate	*Q*_0_	*Q*_0_	Sunshine rate	Sunshine rate	Sunshine rate	*Q*_0_	Sunshine rate	*Q*_0_
2	Sunshine rate	*Q*_0_	Sunshine rate	Sunshine rate	*Q*_0_	*R*_*h*_	*Q*_0_	Sunshine rate	*Q*_0_	Sunshine rate
3	NO_2_	O_3_	*R*_*h*_	*T*_min_	Δ*T*	*Q*_0_	ln(Δ*T*)	*T*_min_	ln(Δ*T*)	*R*_*h*_
4	*T*_min_	*R*_*h*_	ln(Δ*T*)	*R*_*h*_	*R*_*h*_	ln(Δ*T*)	*R*_*h*_	*R*_*h*_	*R*_*h*_	*T*_min_
5	SO_2_	*T*_min_	*T*_max_	NO_2_	O_3_	CO	*T*_min_		PM_10_	*T*_max_
6	PM_10_	SO_2_	PM_2.5_	*T*_max_		PM_2.5_	O_3_		Δ*T*	O_3_
7	AQI	NO_2_	PM_10_	CO		O_3_	NO_2_			NO_2_
8		ln(Δ*T*)		SO_2_						SO_2_
9		*T*_max_		PM_10_						
10				AQI						

Nine city models (model-C) included relative humidity, and based on the analysis of the contribution of meteorological factors to the model, the effects of relative humidity and temperature were equivalent. Urumqi model-C did not include relative humidity, possibly because Urumqi is at the center of the Eurasian continent and experiences a temperate continental arid climate. As can be seen from Table 1, in Urumqi and Lanzhou, Q_0_ and sunshine rate rank first, followed by NO_2_ and O_3_. In Urumqi, NO_2_ ranks third in the impact on solar radiation, and O_3_ ranks third in Lanzhou. The pollution elements selected for model-U were NO_2_, SO_2_, and O_3_, which indicated that there were common air pollutants that affected solar radiation in different cities in China. However, because of dissimilar composition of air pollutants in each city, the selected pollutants in model-C for each city were not the same as model-U. To be specific, five city models included NO_2_, four city models included SO_2_, while four city models included O_3._ In Shenyang and Guangzhou, driving effect of particulate matter (PM_10_ and PM_2, 5_) on the daily global solar radiation was stronger than gas molecules. However, none of the pollution elements in model-C of Shanghai, which differed from the results of other research areas studied previously. This phenomenon may indicate that meteorological factors have a greater impact on solar radiation than pollution factors in Shanghai.

#### City Models (Model-C)

**Figure 5:**
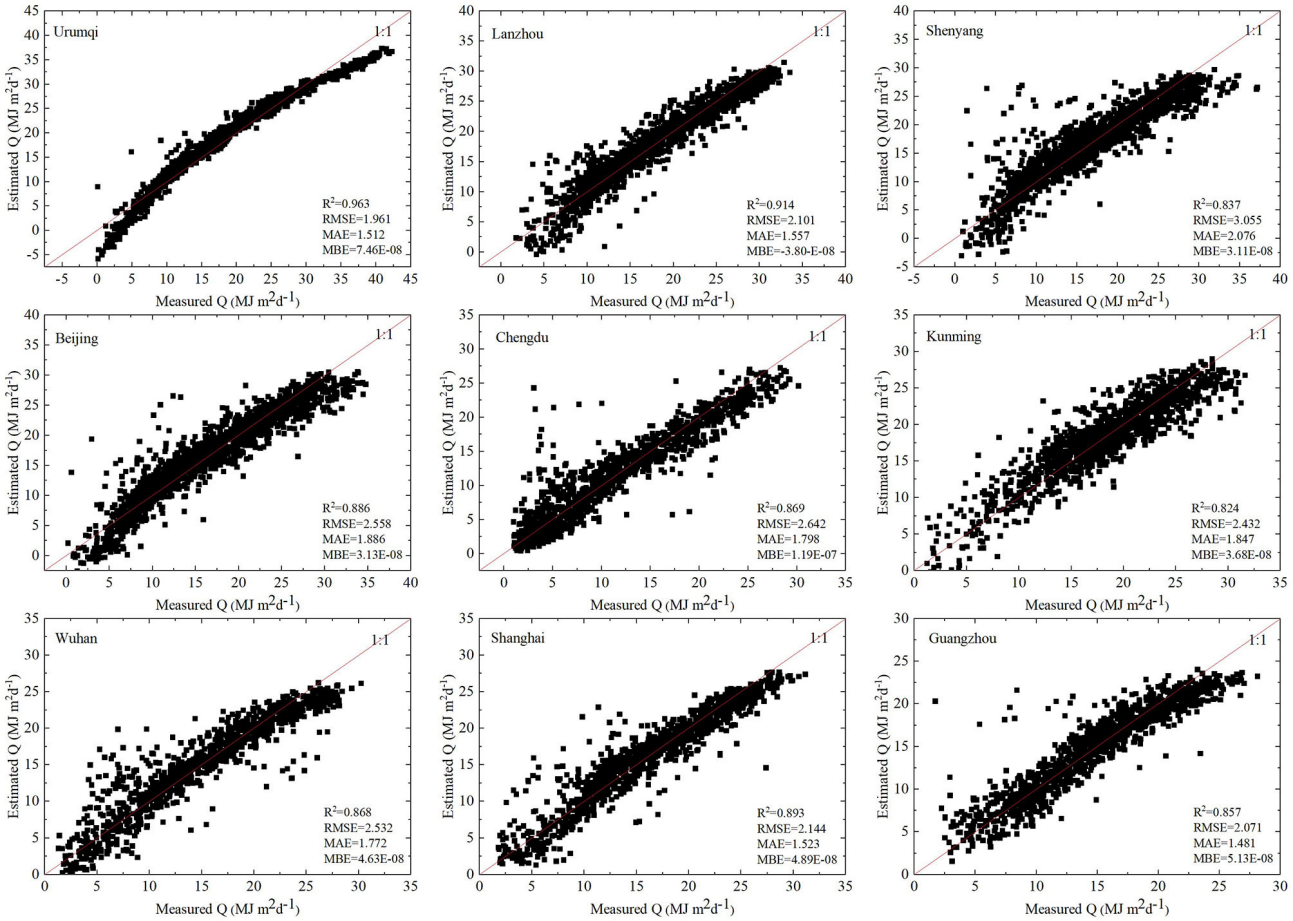
Scatter plot of predicted values and measured values of model-C.

Figure 5 shows the scatter plots of the predicted and measured daily global solar radiation of model-C for each city. The regression results showed that model-C performed well in terms of *R*^2^, RMSE, MAE, and MBE. The restrictive effect of regional pollution factors on solar radiation cannot be ignored. The *R*^2^ of model-C indicated a good fit, suggesting that the solar radiation models based on regional differences can accurately reveal the spatial differences in air pollution. The *R*^2^ value of model-C in Kunming was the lowest (0.824), while that of Urumqi was the highest (0.963), followed by that of Lanzhou (0.914). The *R*^2^ values of the models of other cities ranged between 0.8 and 0.9, indicating that solar radiation of each urban model could be well-explained by the independent variables. Further, the RMSE value of the Shenyang model was the highest (3.055), while that of the Urumqi model was the lowest (1.961). The model RMSE values of other cities ranged mostly between 2 and 3. RMSE initially involves the addition of all errors, which are then squared; therefore, it enlarges the gap between larger errors. MAE can better reflect the actual situation of the predicted value errors. The highest MAE value was observed in Shenyang (2.076), while Guangzhou showed the lowest MAE value (1.481). Further, the mean deviation error of each model-C was low, with that of the Lanzhou model-C being negative, indicating that the predicted value was slightly greater than the measured value. The MBE values of other cities were negative, indicating that the predicted values were lower than the measured values.

#### Unified Models (Model-U)

**Figure 6:**
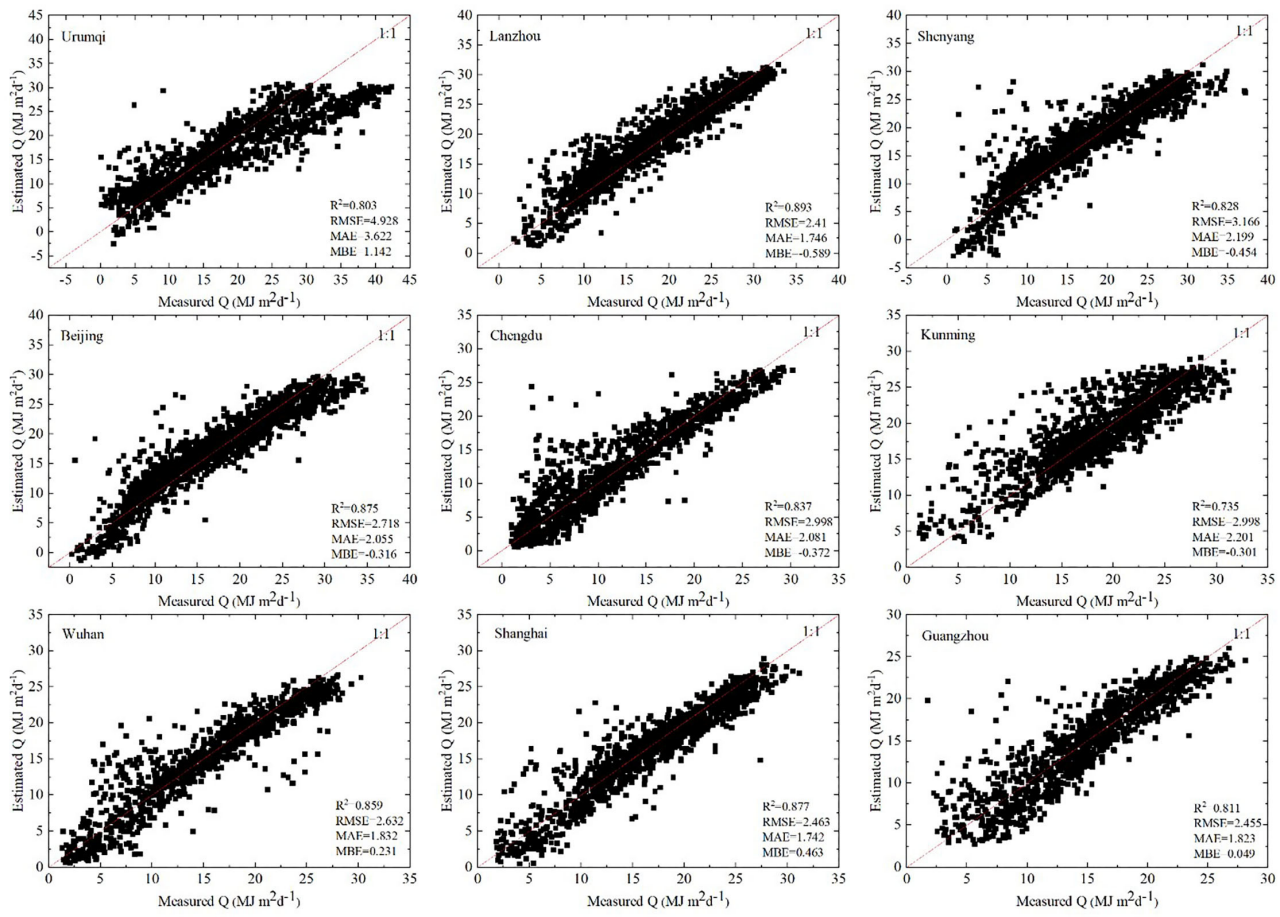
Scatter plot of predicted values and measured values of model-U.

The factors affecting solar radiation as identified by model-U were selected as key pollutants to further simulate and predict solar radiation. Figure 6 shows the scatter plots of the predicted and measured values of model-U for each city. The performance of model-U was the worst in Kunming (*R*^2^ = 0.735), and the best in Lanzhou (*R*^2^ = 0.893). The MBE value of most cities was more than 0, implying that the predicted value was generally less than the measured value, while an MBE value of <0 implied that the predicted value was generally more than the measured value. In general, model-U did not evidently show an overestimation or underestimation tendency. Furthermore, the RMSE (4.928), MAE (3.622), and MBE (1.142) values of Urumqi were the highest among the nine stations. The predicted values of the Urumqi models were underestimated during high solar radiation. In Shenyang, few predicted values were <0, which was inconsistent with the actual scenario. This could be possibly because model-U included maximum and minimum temperatures. When the maximum temperature in winter was <0, the predicted values were negative. In most cities, higher solar radiation values resulted in a better convergence of the scatter plot. However, this does not indicate that a higher actual solar radiation increases the model-U performance. Because model-U considers that precipitation is completely absent, seasons with more rainfall provided less data, which may have also affected the model prediction accuracy. In the future, the issue of low model prediction accuracy during high solar radiation in Urumqi should be addressed to further increase the prediction accuracy of model-U; additionally, negative prediction values in cold winter areas should be addressed, and strategies to improve the model-U accuracy during low solar radiation should be considered.

#### Comparison and Prediction Analysis of Solar Radiation Models

**Figure 7:**
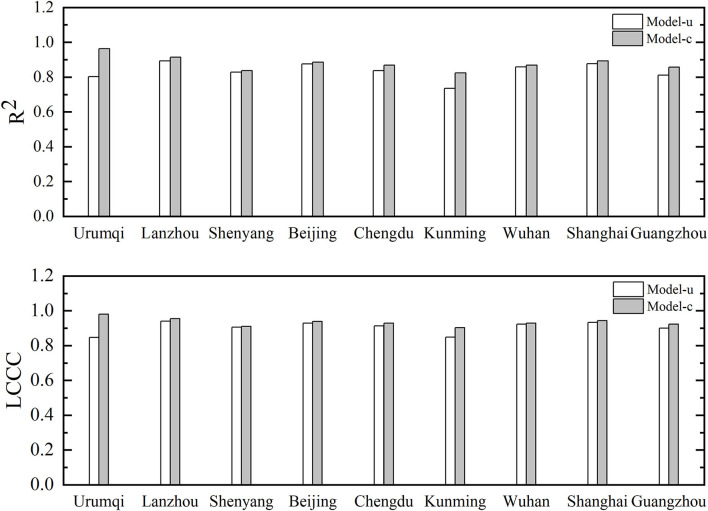
Comparison of *R*^2^ and LCCC values between the two models.

The comparison results of the statistical values of model-U and model-C are shown in Figure 7. The corresponding *R*^2^ values indicated that the prediction performance of model-U in all cities was worse than that of model-C. Further, the model-U prediction performance was not as good as that for the optimal model of each city, and the difference between the performances of the two models was the greatest in Urumqi. Similarly, the LCCC values of the two models differ the most in Urumqi, with model-U being 0.847 and model C being 0.980. The LCCC difference between the two models in Shenyang is the smallest, which is 0.905 and 0.910. In general, except Urumqi, the difference between the two models was not significant in other cities. Additionally, the Urumqi model-U showed a good performance (*R*^2^ = 0.803).

## Discussion

### Influence of Air Pollution on Solar Radiation

Air pollution particles can reduce atmospheric transparency and affect the total solar radiation reaching the ground by reflecting and absorbing solar radiation. After the pollutants in the atmosphere absorb the energy of solar radiation, photochemical reactions may occur to produce toxic substances. Most previous studies analyzed the influence mechanism of AQI on solar radiation. However, this cannot completely reveal the influence mechanism of air pollutants on solar radiation. Therefore, in this study, AQI and six pollutants (PM_2.5_, PM_10_, SO_2_, NO_2_, CO, and O_3_) were considered for mechanism and prediction analyses; subsequently, the mechanism analysis based on pollutants was verified. The comprehensive evaluation index AQI was negatively correlated with solar radiation, while some pollution components (such as O_3_) were positively correlated with the daily total solar radiation. This explains the need and rationality of determining the driving mechanism of atmospheric pollutants on solar radiation in a study area. By adjusting the concentration of various air pollutants, the response mechanism on solar radiation can be realized and the dimming phenomenon in cities can be improved. Further, strategies to assess the impacts of air pollution on solar radiation can be changed from using a conventional comprehensive evaluation index to a comprehensive evaluation method using data on the influence of pollutants on solar radiation. Further, the list of key pollutants is conducive to targeted intervention on air pollutants and can enhance the obtained solar radiation data. In this study, key pollutants were defined as the pollution variables selected for the models during stepwise regression analysis. These pollutants represented the pollution factors that had a strong driving effect on solar radiation in the models. In model-C, the key pollutants evidently differed, and the proportion and types of pollutants in each city model differed. NO_2_ was a key pollutant in five cities, while CO was a key pollutant in only two cities, thus indicating that that the driving effect of NO_2_ was stronger than that of CO in most study areas. The differences in the pollution factors may be caused by the industrial characteristics of cities; however, as various factors affect the daily global solar radiation and the origins of pollutants are complex, further assessments are required. The nine cities selected in this study are almost evenly distributed in China, including four climatic regions. Although evident regional differences were observed in the key pollutants in model-C, some overall similarities were also observed. The key pollutants in model-U were O_3_, NO_2_, and SO_2_, indicating that these were common pollutants affecting the solar radiation in the selected cities. By controlling the emission of key pollutants, the dimming effect in cities can be reduced, which in turn can increase the total solar radiation.

### Prediction Performance of Solar Radiation Models

Based on the *R*^2^, RMSE, MAE, and MBE values of the two models, model-C showed better performance. Local climate and pollution elements were used in model-C, which provided a basis for accurate solar radiation prediction. Model-U used the data on all cities, and showed a similar prediction performance for all nine cities, but the prediction accuracy of each city was worse than that of model-C. Both models showed good prediction performance for solar radiation, with an *R*^2^ value exceeding 0.8. In Urumqi, the difference between the two models was relatively significant, while marginal differences were observed for other cities. This could be possibly because model-U included relative humidity, which was not considered in the Urumqi model-C, thereby causing deviations in the prediction results. This indicates that the accuracy of model-U in arid areas needs to be further verified.

Due to varied industry types and population densities in different cities and different air pollutants in different regions, the driving mechanism of daily global solar radiation differed. Model-C was observed to provide a better prediction accurately using the regional pollution characteristics and the impact of key pollutants on solar radiation than only using meteorological factors. Simultaneously, controlling the emission of key pollutants can allow the coordinated development of air quality and solar energy resource utilization during urbanization. Although the model-U performance was not as good as that of model-C, it still showed high prediction accuracy. Model-U can be applied in areas lacking long-term solar radiation monitoring data in China to obtain relatively accurate prediction data.

### Limitations and Future Work

This study has some limitations. First, problems in data acquisition limited the regional coverage of the study. Some areas, such as plateau areas, were not included in the study. In addition, factors such as topography, wind and climate may affect pollutant diffusion in the stratosphere, thus adding more uncertainty and complexity to the regional solar radiation prediction and photochemical reaction. This highlights the need to consider the impact of the lag effect of air pollution on solar radiation. Second, due to the coupling effect of many factors, comprehensively analyzing the driving effect of various pollutants on solar radiation is difficult. Thus, the driving mechanism of individual pollutants should be further studied. The analysis results of driving mechanism indicated that several problems need to be resolved to alleviate the negative impacts of air pollution on solar radiation. Despite these limitations, this study effectively evaluated the correlation between different air pollutants and solar radiation, and identified key pollutants that have a strong impact on solar radiation in each study area; additionally, key pollutants suitable for the national prediction model were shortlisted, which also can be used as a reference for other regions.

## Conclusion

Many studies have indicated that the impact of air pollution on solar radiation cannot be neglected. Previous studies mostly analyzed the impact of AQI on solar radiation through a general perspective of air pollution; however, they ignored the differences in the impacts of different pollutants (PM_2.5_, PM_10_, NO_2_, SO_2_, CO, and O_3_) on solar radiation. Because direct accurate measurement of solar radiation data is difficult, solar radiation prediction models are gaining increasing importance. Understanding the differences in the impacts of pollutants on solar radiation is significant to improve the model prediction accuracy and formulate effective pollution control policies. In this study, Pearson's correlation coefficient was used to analyse the correlation between different pollutant types and daily global solar radiation, while the influence mechanism of air pollutants on daily total solar radiation was analyzed using stepwise regression. Varying industrialization levels and climate conditions in different cities can result in different impacts of air pollutants on solar radiation in different regions. The key pollutants that influence solar radiation were identified by model-U and model-C. The accuracy of model-C was higher than that of model-U, but model-U is significant for areas lacking long-term solar radiation data. Further, the key pollutants reflect the regional heterogeneity of the impacts of air pollution on solar radiation, which can assist in improving the model accuracy, proposing more targeted pollution control countermeasures, and promoting the efficient utilization of solar energy resources.

## Data Availability Statement

Publicly available datasets were analyzed in this study. This data can be found at: China National Environmental Monitoring Center; http://www.cnemc.cn/, China National Meteorological Science Data Center; http://data.cma.cn/Market/Detail/code/RADI_CHN_MUL_HOR/type/0.html.

## Author Contributions

XZ: conceptualization, formal analysis, and writing—original draft. MZ: data curation and software. YC: formal analysis and visualization. YH: methodology and writing—review and editing. All authors contributed to the article and approved the submitted version.

## Funding

This research project was sponsored by the National Natural Science Foundation of China (Grant no. 51878089).

## Conflict of Interest

MZ was employed by Chongqing Real Estate Trading Centre, Chongqing, China. The remaining authors declare that the research was conducted in the absence of any commercial or financial relationships that could be construed as a potential conflict of interest.

## Publisher's Note

All claims expressed in this article are solely those of the authors and do not necessarily represent those of their affiliated organizations, or those of the publisher, the editors and the reviewers. Any product that may be evaluated in this article, or claim that may be made by its manufacturer, is not guaranteed or endorsed by the publisher.
